# Synaptic Transistors Based on PVA: Chitosan Biopolymer Blended Electric-Double-Layer with High Ionic Conductivity

**DOI:** 10.3390/polym15040896

**Published:** 2023-02-10

**Authors:** Dong-Hee Lee, Hamin Park, Won-Ju Cho

**Affiliations:** 1 Department of Electronic Materials Engineering, Kwangwoon University, Gwangun-ro 20, Nowon-gu, Seoul 01897, Republic of Korea; 2 Department of Electronic Engineering, Kwangwoon University, Gwangun-ro 20, Nowon-gu, Seoul 01897, Republic of Korea

**Keywords:** organic synaptic transistor, PVA, chitosan blended, biopolymer, biocompatible, electric-double-layer, neuromorphic computing system

## Abstract

This study proposed a biocompatible polymeric organic material-based synaptic transistor gated with a biopolymer electrolyte. A polyvinyl alcohol (PVA):chitosan (CS) biopolymer blended electrolyte with high ionic conductivity was used as an electrical double layer (EDL). It served as a gate insulator with a key function as an artificial synaptic transistor. The frequency-dependent capacitance characteristics of PVA:CS-based biopolymer EDL were evaluated using an EDL capacitor (Al/PVA: CS blended electrolyte-based EDL/Pt configuration). Consequently, the PVA:CS blended electrolyte behaved as an EDL owing to high capacitance (1.53 µF/cm^2^) at 100 Hz and internal mobile protonic ions. Electronic synaptic transistors fabricated using the PVA:CS blended electrolyte-based EDL membrane demonstrated basic artificial synaptic behaviors such as excitatory post-synaptic current modulation, paired-pulse facilitation, and dynamic signal-filtering functions by pre-synaptic spikes. In addition, the spike-timing-dependent plasticity was evaluated using synaptic spikes. The synaptic weight modulation was stable during repetitive spike cycles for potentiation and depression. Pattern recognition was conducted through a learning simulation for artificial neural networks (ANNs) using Modified National Institute of Standards and Technology datasheets to examine the neuromorphic computing system capability (high recognition rate of 92%). Therefore, the proposed synaptic transistor is suitable for ANNs and shows potential for biological and eco-friendly neuromorphic systems.

## 1. Introduction

The recent rapid increase in the amount of unstructured data has highlighted the importance of information processing capabilities in the field of artificial intelligence, including big data [1,2,3]. However, in computing systems such as CPUs and GPUs with von Neumann architectures, information is processed and stored separately based on data and commands from memory. Consequently, when processing large amounts of data and performing artificial intelligence algorithms, the von Neumann bottleneck leads to performance limitations such as slow processing and high-power consumption between the computing units and data storage [4,5,6]. Thus, neuromorphic semiconductor technology, which replaces the Boolean logic and information processing method of the conventional von Neumann structure, has garnered attention owing to its efficient processing of information using a computing system that imitates the human brain. The human brain, which comprises approximately 100 billion neurons and 100 trillion synapses, can process and store large amounts of data quickly through large-scale parallel computations even with a low power of 20 W [7,8]. Neuronal synapses in pathways between pre-neuron and post-neuron, which are the biological functions responsible for transmitting, processing, and storing information in the brain, are the primary parts where spike currents occur. Synaptic plasticity results from changes in synaptic weight that can be modulated through the flow of electrical currents and mobile protonic ions through the synapse [9,10,11]. Therefore, a synaptic transistor that can implement synaptic plasticity as a key component of a neuromorphic chip mimicking a high-efficiency system of the brain is being actively studied [12,13]. The synaptic transistor consumes relatively little power and can reliably implement spike generation and electrical plasticity. In addition, various neuron synaptic behaviors can be accurately emulated through the activation of the ion channels of post-synaptic neurons that occur during stimulation transfer and the efficient control of channel conductivity [14,15,16]. In particular, a synaptic transistor with a polymer electrolyte as an insulating layer imitates a synaptic function while resulting in an electrical double layer (EDL) effect at each interface (electrode/electrolyte, electrolyte/channel). The EDL transistor exhibits a high capacitance value (>1 µF/cm^2^) owing to the EDL effect based on internal mobile protonic ions [17,18,19]. Further, they are used as synaptic devices through the accumulation of high-density charges even at low voltages owing to the polarization reaction of mobile protonic ions. Thus, they are expected to be eco-friendly biomaterials, leading to low power consumption. Accordingly, with the increasing importance of bio-functional electronic devices, studies on eco-friendly materials have been actively conducted. In particular, diverse organic materials such as polyvinyl alcohol (PVA), milk, chitosan (CS), egg, albumin, and cellulose have been used as electrolytes to develop biocompatible EDL synaptic transistors. Such transistors are biodegradable, non-toxic, biocompatible, suitable for bio-friendly electronic systems, and naturally abundant, enabling cost-effective processing [20,21].

This study proposed a synaptic transistor based on PVA:CS blended EDL, a biocompatible polymer material with high ion conductivity. Among various polymer electrolytes, biodegradable polymers are preferable owing to their renewability and biocompatibility. In particular, PVA and CS are highly valuable environmentally friendly biodegradable polymers. PVA exhibits excellent properties such as high chemical stability, hydrophilicity, safety, and low power consumption; CS is non-toxic, cost-effective, and has a large charge storage capacity. In addition to their respective features, blending these two polymers can increase the ionic conductivity and diminish the crystallinity of the polymer. Therefore, it has been widely used in various device applications such as supercapacitors, solar cells, and batteries [22,23,24]. The EDL effect based on the proton charges of the PVA:CS blending electrolyte was verified through the evaluation of the frequency-dependent capacitance characteristics using an EDL capacitor with an Al/PVA:CS blended electrolyte-based EDL/Pt configuration. Electronic synaptic transistors were manufactured by using indium-tin-oxide (ITO), PVA:CS blended electrolyte based-EDL, and indium-gallium-zinc oxide (IGZO) as the bottom gate electrode, gate insulator, and channel, respectively. Subsequently, the ITO gate and the IGZO channel performed pre-synapse and post-synapse roles, respectively, and the mobile ions in the PVA:CS blended electrolyte-based EDL were used as neurotransmitters. Through evaluations of the transfer and output characteristics of the prepared synaptic transistor, the PVA:CS blended EDL successfully implemented synaptic behavior based on the polarization reaction of mobile protonic ions. In addition, fundamental artificial synaptic behaviors such as hysteresis, single-spike excitatory post-synaptic current (EPSC), paired-pulse facilitation (PPF), dynamic signal filtering, and spike-timing-dependent plasticity (STDP) were successfully evaluated. Furthermore, potentiation/depression characteristics were successfully implemented in repeated cycles by using PVA:CS blended EDL. Finally, the applicability of the proposed synaptic transistor to neuromorphic systems was demonstrated through learning simulations and recognition of the Modified National Institute of Standards and Technology (MNIST) handwritten digits using an artificial neural network (ANN) model.

## 2. Materials and Methods

### 2.1. Materials 

Corning 7509 glass substrate (Corning Inc., New York, NY, USA), chitosan powder (deacetylation degree > 75%, Sigma Aldrich, Seoul, Republic of Korea), PVA powder (technical grade, Sigma Aldrich, Seoul, Republic of Korea), acetic acid solution (purity > 99%, Sigma Aldrich), IGZO sputter target (In_2_O_3_:Ga_2_O_3_:ZnO = 4:2:4.1 mol%, THIFINE, Incheon, Korea), ITO sputter target (In_2_O_3_:SnO_2_ = 9:1 mol%, THIFINE), and Al pellets (purity > 99%, TFN, Seoul, Republic of Korea) were used for this study. 

### 2.2. Preparation of PVA:CS Blended Solution 

**Figure 1:**
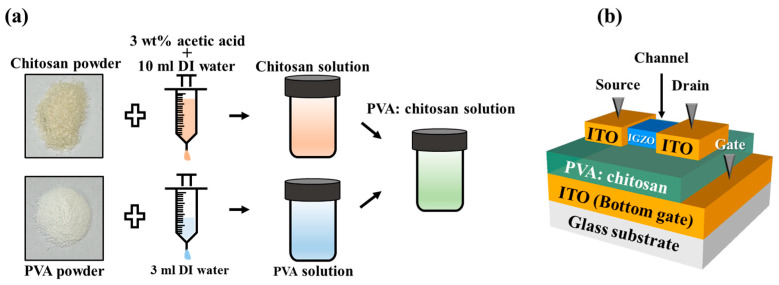
(**a**) Fabrication details of PVA:CS blended solution. (**b**) Schematic diagram of PVA:CS biopolymer blended EDL-based synaptic transistor on glass substrate.

A biocompatible PVA:CS blended solution was prepared by mixing a PVA solution and a chitosan solution, respectively. A PVA solution was synthesized by dissolving a mixture of 10 wt% PVA powder and 3 mL diluted deionized (DI) water. In addition, a chitosan solution was synthesized by dissolving a mixture of 2 wt% chitosan powder based on the shrimp shell, 2% acetic acid solution, and 10 mL DI water. The PVA solution and chitosan solution were completely dissolved using a magnetic stirrer with 600 rpm at 90 °C for 2 h and 800 rpm at 50 °C for 6 h, respectively. The solutions were then filtered using a 5 μm pore size polytetrafluoroethylene (PTFE) syringe filter (Whatman, International Ltd., Maidstone, UK) to remove impurities. Finally, the two solutions were completely mixed in a 1:1 ratio using a magnetic stirrer at 800 rpm at room temperature for 6 h. Figure 1a shows the fabrication details of the PVA:CS blended solution.

### 2.3. Fabrication of EDL Synaptic Transistors Based on PVA:CS Biocompatible Blended Electrolyte

Figure 1b shows the schematic of the PVA:CS biocompatible blended electrolyte-based EDL synaptic transistor. A fully transparent Corning 7509 glass substrate was cleaned considering the Standard Radio Corporation (RCA) to remove particles. Subsequently, an ITO film with a thickness of 300 nm was deposited on a glass substrate to form a bottom gate electrode using radio frequency (RF) magnetron sputtering with an Ar gas flow rate of 20 sccm, RF power of 100 W, and operating pressure of 3.0 mTorr. The prepared PVA:CS blended solution, which is crucial to the high ionic conductivity EDL of synaptic transistors, was spin-coated on the bottom gate electrode at 4000 rpm for 50 s and then dried in the air at room temperature for 24 h. Subsequently, an IGZO channel layer with a thickness of 50 nm was deposited by RF magnetron sputtering with an Ar gas flow rate of 30 sccm, an RF power of 100 μm, and an operating pressure of 6.0 m Torr through a shadow mask. The channel length (L) and width (W) of the prepared synaptic transistor were 80 and 1000 μm, respectively. Finally, an ITO layer with a thickness of 150 nm was deposited as source and drain (S/D) electrodes with dimensions of 200 µm × 1000 µm through a shadow mask using RF magnetron sputtering. 

### 2.4. Characterization 

We used Fourier transform infrared (FTIR) spectroscopy (IFS66v/s & Hyperion3000, Bruker Optiks, Germany), a fast and general analytical technique widely used to analyze chemical compounds, to analyze the chemical group and molecular structure of the PVA:CS biopolymer blended EDL. Further, The Al/PVA:CS blended electrolyte-based-EDL/Pt capacitors were fabricated to measure the frequency-dependent capacitance characteristics using an Agilent 4284A Precision LCR meter (Agilent Technologies, Santa Clara, CA, USA). The electrical characteristics of proposed synaptic transistors were measured using an Agilent 4156B Precision Semiconductor Parameter Analyzer (Hewlett-Packard Co., Palo Alto, CA, USA). In addition, the synaptic operation was investigated through the application of pre-synaptic and post-synaptic pulse using an Agilent 8110A pulse generator (Hewlett-Packard Co.). While measurements were taken, the synaptic transistors were placed on the probe station in a dark shielded box to prevent electrical noise and external light.

## 3. Results and Discussion

### 3.1. Characteristics of PVA:CS Biopolymer Blended EDL Membrane

**Figure 2:**
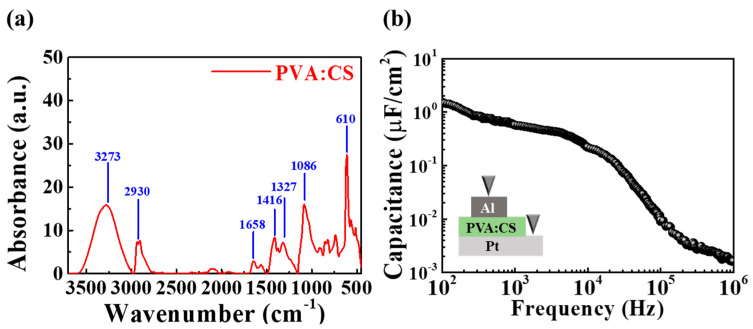
(**a**) FTIR spectra of PVA:CS blended electrolyte films. (**b**) Frequency–dependent capacitance (C–*f*) curve of Al /PVA:CS blended electrolyte-based EDL/Pt-structured capacitors.

To predict the electrical properties and synaptic behavior of the EDL transistor having the PVA:CS biopolymer as a gate insulator, the chemical composition of the PVA:CS blended electrolyte film was analyzed following sufficient drying in after spin coating by FT-IR. Figure 2a shows the FT-IR spectrum of the PVA:CS blended electrolyte film in the wavelength range of 3700–450 cm^−1^, where the wide peaks at 3273 and 2930 cm^−1^ were owing to O-H and C-H stretching, respectively. The range from 1700–1600 cm^−1^ was dominated by the amide I region, and the peak at approximately 1658 cm^−1^ was owing to C=O stretching. In addition, the range from 1500–1300 cm^−1^ represents the amide II region, and equivalent peaks at approximately 1416 and 1327 cm^−1^ were owing to N-H bending and C-N stretching, respectively. Further, the peaks at approximately 1086 and 610 cm^−1^ were owing to C-OH stretching and C-H vibrations, respectively [25,26,27,28]. Consequently, peaks based on the amide and -OH groups were commonly observed in the FT-IR spectra of PVA:CS blended electrolyte films. Furthermore, those associated with the -OH group contributed to the mobility and conductivity of protons [29,30]. Thus, ion conduction occurs because of mobile protonic ions in the PVA:CS blended electrolyte upon the application of a voltage, and EDL may be formed at an interface of the PVA:CS blended electrolyte.

The EDL with strong coupling effects and high capacitance enables high-efficiency artificial synapse construction through the constant accumulation of high-density proton charges that control ionization and conductivity [31]. Accordingly, an EDL capacitor with an Al/PVA:CS blended electrolyte-based EDL/Pt structure was prepared to verify the EDL effect of PVA:CS blended electrolyte, and the frequency-dependent capacitance characteristics were evaluated. Figure 2b shows the capacitance change measured over a frequency range of 100 Hz to 1 MHz. With the gradual decrease in the frequency from 1 MHz, the capacitance increased, yielding a high capacitance value of 1.53 μF/cm^2^ at 100 Hz. The frequency-dependent response of the mobile protonic ions affected the change in capacitance. As the response time is not sufficient at high frequencies, the mobile protonic ions encounter difficulties when attempting to reach the interface, resulting in low capacitance. However, the capacitance is high at low frequencies because the mobile protonic ions accumulate at the interface, owing to the response time being sufficient to form the EDL. Consequently, the EDL effect in the PVA:CS blended electrolyte became apparent as a frequency-dependent capacitance characteristic leading to large capacitance [32]. This verified that the proposed PVA:CS blended EDL membrane was applicable to EDL-based synaptic devices.

### 3.2. Electrical Characteristics of Synaptic Transistors Based on PVA:CS Biopolymer Blended EDL with High Ionic Conductivity

**Figure 3:**
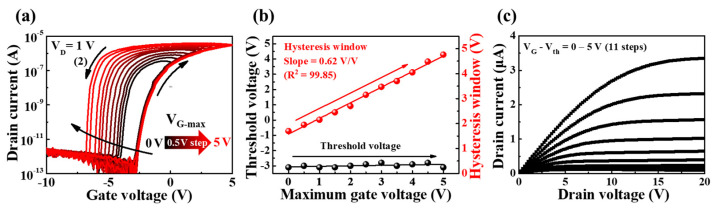
(**a**) Transfer characteristic (I_D_–V_G_) curves as a function of maximum gate voltage (0 to 5 V in 0.5 V steps) at constant V_D_ = 1 V in double sweep mode. (**b**) Hysteresis window and threshold voltage extracted from transfer curves in double sweep mode. (**c**) Output characteristic (I_D_–V_D_) curves measured at V_G_–V_th_ from 0 to 5 V at 0.5 V increments.

Figure 3a illustrates the transfer characteristic (I_D_–V_G_) curves in double sweeps of gate voltage (*V_G_*) of a PVA:CS biopolymer blended EDL synaptic transistor with high ion conductivity. The transfer curves were measured at a constant drain voltage (V_D_) of 1 V, with maximum gate voltage sweep increasing positively (forward) from 0 to 5 V in 0.5 V steps and then returning negatively (reverse). It can be confirmed that counterclockwise hysteresis appears in the V_G_ double sweep mode of transfer curves, and the hysteresis window widens when the V_G-max_ sweep range is increased. This counterclockwise hysteresis phenomenon implies a slow polarization reaction by mobile protonic ion migration in PVA:CS blended electrolyte-based EDL. With an increase in the maximum V_G_ in the forward sweep, more mobile protonic ions accumulated at the interface between the IGZO channel and the PVA:CS blended electrolyte, thereby resulting in a stronger electric field. In contrast, in a reverse sweep, protons moved steadily in the opposite direction, which increased the counterclockwise hysteresis. Figure 3b presents the hysteresis window and threshold voltage (V_th_) extracted from the double sweep transfer curves for V_G-max_. With an increase in V_G-max_ from 0 to 5 V at 0.5 V intervals, the hysteresis window increased in a linear manner from 1.57 to 4.71 V with high linearity (R^2^) of 99.85 and a slope of 0.62 V/V; however, V_th_ remained approximately constant at −3.05 V. Figure 3c shows the measured output characteristic (I_D_-V_D_) curves at V_G_-V_th_ values from 0–5 V in steps of 0.5 V. With an increase in V_D_, the drain current (I_D_) increased in a linear manner and gradually saturated, exhibiting a typical pinch-off characteristic.

### 3.3. Synaptic Characteristics of PVA:CS Blended Electrolyte-Based EDL Synaptic Transistors

**Figure 4:**
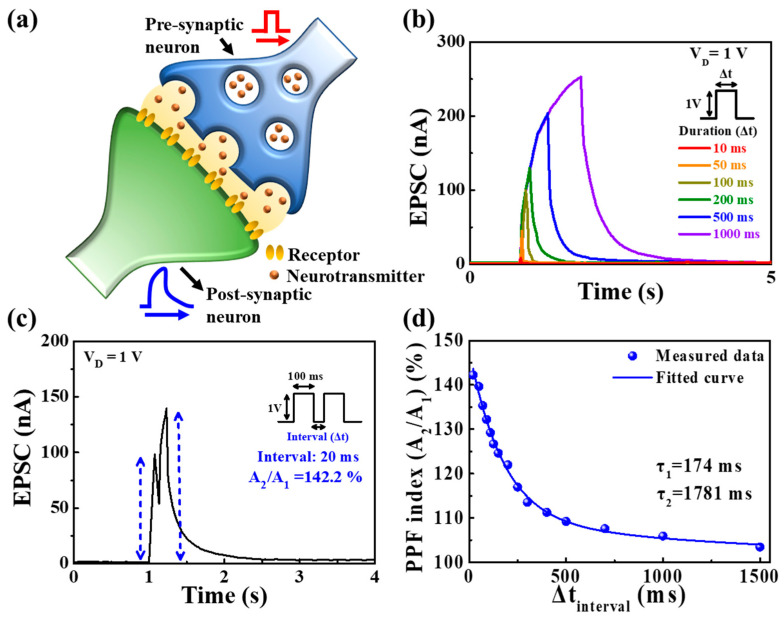
(**a**) Schematic structure representing biological synapses in the actual brain. (**b**) EPSC responses to pre-synaptic single spike pulses with amplitude of 1 V at different durations (10, 50, 100, 200, 500, and 1000 ms). (**c**) EPSCs triggered by paired pre-synaptic spikes (1 V, 100 ms) at time interval (Δ*t*_interval_) = 20 ms. (**d**) PPF index (A_2_/A_1_) for different Δ*t*_interval_ at a spike time of 20–1500 ms.

Figure 4a illustrates a schematic structure representing biological synapses in the actual brain. To implement synaptic transistors, synaptic behavior that mimics the structure and mechanism of biological synapses is essential. Synapses that play an important role in signal transmission include nanogaps between pre-synaptic and post-synaptic neurons. Due to the diffusion of neurotransmitters in the synapse, the pre-synaptic neuronal signal spikes are conveyed to the post-synaptic neurons [33,34]. In the prepared synaptic transistor, electrical spikes applied to the ITO bottom gate (pre-synapse) were delivered to the IGZO channel (post-synapse) via a PVA:CS blended electrolyte-based EDL (neurotransmitter). Channel current evoked by electrical spikes in the pre-synaptic gate is expressed as EPSC and is a fundamental representation of synaptic strength. Figure 4b shows single-spike EPSC retention curves as a function of the pulse duration (10 to 1000 ms) of a pre-synaptic stimulus spike with a pulse amplitude of 1 V (V_D_ = 1 V). The longer the pre-synaptic spike duration, the higher the maximum EPSC, indicating an increase in EPSC retention time. Further, the current in the post-synaptic channel increased the duration of the pre-synaptic gate pulse. This indicates that the longer the spike duration, the greater the number of ions migrating to the PVA:CS mixed electrolyte/channel interface, which results in a higher concentration gradient and channel conductance. Therefore, the proposed PVA:CS electrolyte-based EDL synaptic transistor implemented synaptic plasticity modulation according to channel conductance via electrical spike stimulation.

In neuroscience, neural facilitation is a dynamic enhancement of the transporter and reflects the receptivity of biological synapses for information processing. PPF is an important property of short-term synaptic plasticity wherein the first post-synaptic potential or current is amplified by the second pre-synaptic spike depending on the spike time interval (Δ*t*_interval_) [21,35]. In the EDL layer, the mobile protonic ions are transported between the interface and the electrolyte by the first pre-synaptic spike, which continues to accumulate on the interface owing to the lack of time to return to their initial position at a short Δ*t*_interval_. Figure 4c shows EPSCs triggered by two consecutive pre-synaptic spikes (1 V, 100 ms) with a spike interval time of 20 ms. In the case of a short interval time, the time for mobile ions to return to their original positions is insufficient, which results in the continuous accumulation of mobile protonic ions at the interface. These incompletely relaxed mobile protonic ions influence the first pre-synaptic spike and consequently evoke a larger EPSC peak at the second spike. Figure 4d shows the PPF index (A_2_/A_1_) extracted by the ratio of the amplitude of the two EPSCs peak as a function of various spike pulse interval times [36]. The PPF index (A_2_/A_1_) exhibited a high value of ~142.2% at Δ*t*_interval_ = 20 ms; however, it gradually decreased to 103.5% when the spike time interval was sufficiently long (1500 ms). The measured data can be fitted with a double exponential decay function as follows [21].

(1) $$PPF\;index=A+C_{1}\exp(-\Delta t/\tau_{1})+C_{2}\exp(-\Delta t/\tau_{2})$$

where $\tau_{1}$ and $\tau_{2}$ are the relaxation times of the respective phases and *C*_1_ and *C*_2_ are the initial facilitations magnitudes. The relaxation time constants $\tau_{1}$ and $\tau_{2}$ in the proposed synaptic transistor were 174 and 1781 ms, respectively, comparable to those of biological synapses [37,38].

**Figure 5:**
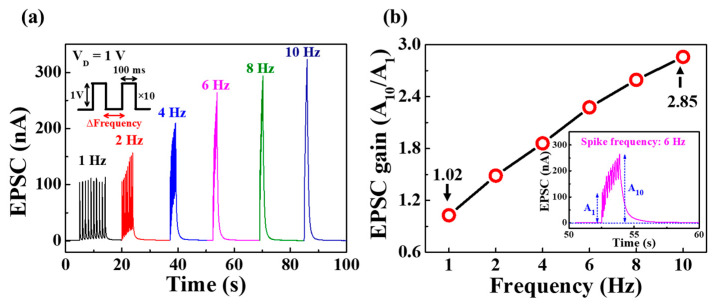
(**a**) EPSC response to consecutive pre-synaptic spikes (1 V, 100 ms) at different frequencies (1 to 10 Hz). (**b**) EPSC gain (A_10_/A_1_) value. The EPSC response to 6 Hz is shown inset.

Synaptic transistors can also act as dynamic filters for information transfer depending on changes in signal frequency, with short-term synaptic facilitation and depression contributing to high-pass and low-pass temporal filtering, respectively [39]. Figure 5a shows the EPSC responses triggered by 10 consecutive pre-synaptic spikes (amplitude 1 V, duration 100 ms) at various frequencies from 1 to 10 Hz, with the EPSC peak value increasing with extending frequency. At a relatively low frequency of 1 Hz, the EPSC response triggered by each spike remained at 113.28 nA, similar to the maximum EPSC value triggered by the first spike. On the other hand, it was confirmed that the EPSC peak value increased due to the gradual increase in frequency. Figure 5b illustrates the EPSC gain values (A_10_/A_1_) at different frequencies. The EPSC gain is calculated as a ratio of the tenth EPSC (A_10_) to the first EPSC (A_1_). As the frequency increases from 1 Hz to 10 Hz, the EPSC gain also increases from 1.02 to 2.85, demonstrating that the proposed synaptic transistor can successfully perform as a high-pass filter for processing information. 

**Figure 6:**
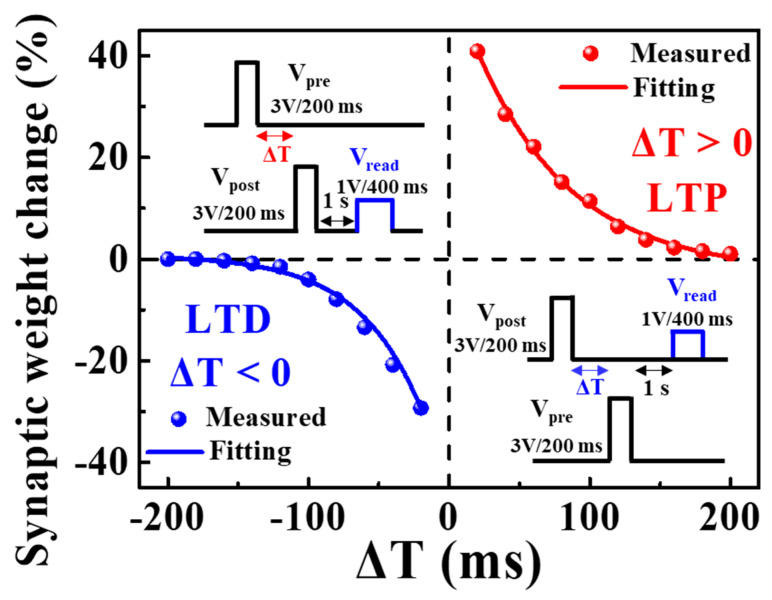
STDP characteristics for excitatory response modes. Inset represents the sequence of spikes between pre–synaptic and post-synaptic signals according to their relative timing differences.

Contrary to short-term plasticity, long-term plasticity, which represents long-term changes in synaptic weights, creates storage of information in a neural system. Relatively monotonous and fast-transmitted stimuli can strengthen the synaptic weight. Long-term potentiation (LTP) indicates a continuous increase in synaptic weight, while long-term depression (LTD) refers to a long-term weakening of synaptic weight. For the proposed synaptic transistor, the spike time of both LTP and LTD was identified as STDP, an important mechanism for learning and memory functions in the human brain [40,41]. Furthermore, with improved Hebbian learning rules that consider the temporal sequence of activities between pre-synaptic and post-synaptic neurons, STDP controls the strength of connections between various neurons through temporally corresponding neural learning [42,43,44]. Figure 6 shows the STDP characteristics for the excitatory response mode. The synaptic weight relative to the timing difference (Δ*T* = *t*_post_ − *t*_pre_) was affected by the pre-synaptic arrival time (*t*_pre_) and post-synaptic production time (*t*_post_). The synaptic weight change (Δ*W*) was calculated using Equation (2) as follows [45]:

(2) $$\Delta W=\frac{I_{\mathrm{post}}-I_{\mathrm{pre}}}{I_{\mathrm{pre}}}\times 100\%$$

where *I*_post_ and *I*_pre_ are the drain currents measured with drain pre-read and post-read pulses, respectively, with 1 V amplitude and 400 ms duration. The calculated result of ΔW shows the property of the asymmetric Hebbian learning STDP [46]. With the decrease of |∆*T*|, the change in synaptic weight was strengthened while the pre-synaptic spike was ahead of the post-synaptic spike (∆*T* > 0), resulting in LTP characteristics. In contrast, when the post-synaptic spike occurred prior to the pre-synaptic spike (Δ*T* < 0), the synaptic weight was weakened, resulting in LTD characteristics. Moreover, when |∆*T*| increased, the synaptic weight change diminished. The extracted ΔW can be well-fitted through the application of the STDP learning function defined by the following equation [43].

(3) $$\Delta W=\{\begin{array}{c} A^{+}e^{-\Delta T/\tau^{+}},\;\;if\;\Delta T>0\\ -A^{-}e^{\Delta T/\tau^{-}},\;\;if\;\Delta T<0 \end{array}$$

where τ^±^ is the time constant representing the range of Δ*T* over which synaptic connections are strengthened and weakened, respectively. The maximum synaptic weight change that occurred when *T* approached zero was determined by *A*^+^ and *A*^−^. These results demonstrate the ability of the proposed PVA:CS biopolymer blended electrolyte-based EDL synaptic transistor to emulate biological STDP properties. 

**Figure 7:**
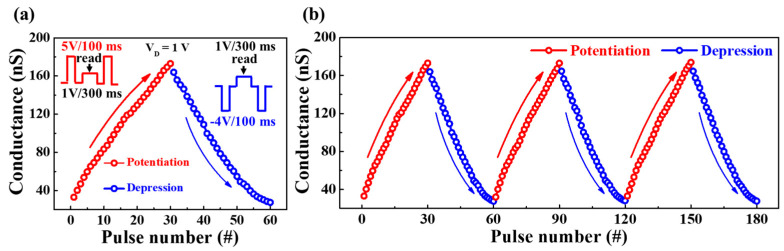
(**a**) Channel conductance modulation by application of 30 potentiation pulses and 30 depression pulses to the pre-synapse. (**b**) Endurance characteristics for three consecutive cycles of potentiation and depression.

Subsequently, to understand the gradual conductance modulation according to the electrical pulse stimulation, the increase or decrease of the synaptic weight was investigated. Figure 7a shows the channel conductance modulation of potentiation and depression of repetitive pre-synaptic pulses. The insets illustrate the conditions of potentiation or depression read pulse for a single pre-spike. One cycle of pre-synaptic pulses comprised 30 potentiation pulses (5 V/100 ms) and 30 depression pulses (−4 V/100 ms) applied to the bottom gate electrode. In addition, a read pulse of 1 V was applied to the drain for 300 ms to measure the change in channel conductance owing to the pre-synaptic pulse. Further, the dynamic range of channel conductance was 32.9 to 172.9 nS for the proposed synaptic transistor. Figure 7b illustrates the endurance of the successive conductance when the potentiation and depression modulations in (a) were repeated for three cycles. As evident, the conductance modulation remained substantially constant. Thus, these results demonstrate the applicability to future artificial synaptic transistors by indicating that the LTP/LTD properties of the proposed device evoked by repetitive pulses and the learning process can be performed in an ANN.

### 3.4. MNIST ANN Simulation of Proposed Synaptic Transistors

**Figure 8:**
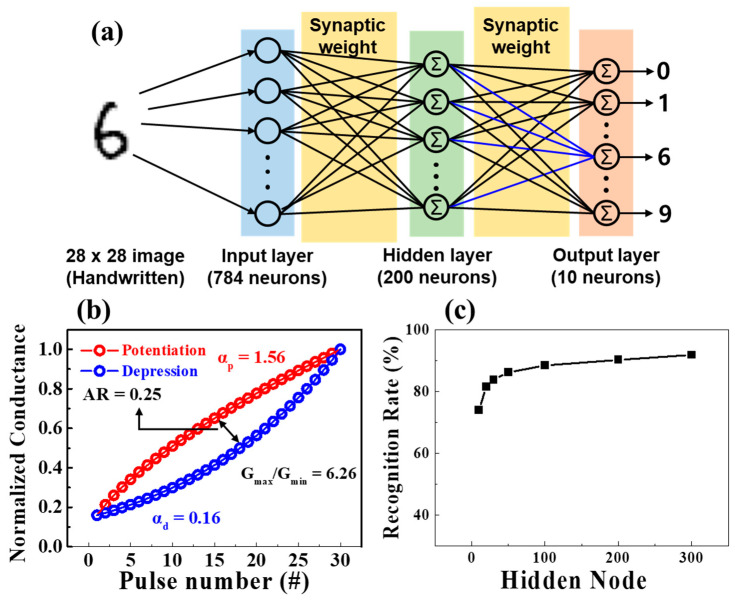
(**a**) Schematic diagram of a three-layer connected ANN with input, hidden, and output layers for MNIST recognition. (**b**) Nonlinearity analysis for normalized potentiation and depression (*G*/*G_max_*). (**c**) Simulated recognition rates with various numbers of hidden nodes in epoch 1.

To verify whether the proposed synaptic transistor exhibited neuromorphic computing capabilities, the MNIST handwritten digit learning was simulated using a three-layer perceptron network model. Figure 8a shows the designed ANN comprising 784, 200, and 10 neurons in the input, hidden, and output layers, respectively. The neurons constituting the input and output layers correspond to 28 × 28 pixels and the numbers 0 to 9 of the binarized MNIST data, respectively. As shown in Figure 8b, each neuron was connected to another via a synapse, and the connection strength was related to the normalized potentiation and depression conductivity of the PVA:CS blended electrolyte-based EDL synaptic transistor. For the MNIST pattern recognition simulations, we used the normalized potentiation and depression properties of the proposed synaptic transistor. The normalized conductance was extracted by dividing each conductivity by the maximum conductivity (*G/G_max_*) in Figure 7a. Additionally, dynamic range (DR), asymmetric ratio (*AR*), and linearity have significant roles in the accuracy of learning and recognition models in potentiation and depression. The DR representing the conductance modulation range defined by *G_max_/G_min_*, was 6.26. The recognition rate depends on this value. Normally, a lower DR results in lower performance, and thus a higher DR value is required to improve accuracy [47]. The *AR* is expressed as a ratio of conduction modulation asymmetry. The following equation was used for extracting *AR*, where *G_p_*(n) and *G_d_*(n) indicate conductance values according to the n^th^ pulse of potentiation and depression, respectively [48].

(4) $$AR=\frac{MAX\left|G_{p}\left(n\right)-G_{d}\left(n\right)\right|}{G_{p}\left(30\right)-G_{d}\left(30\right)}\;for\;n=1\;to\;30$$

When the *AR* approached 0, the training accuracy increased, thus approaching the ideal learning scenario. The calculated *AR* value of the proposed synaptic transistor was 0.25, which is close to the ideal value. In addition, to identify the linearity of the conductance, the nonlinearity coefficient was calculated using the following equation [49].

(5) G={{(Gmaxα−Gminα)× w+Gminα}1αGmin×(Gmax/Gmin)w      if α≠0,      if α=0.

where *G_max_* and *G_min_* represent the maximum and minimum conductivity values, respectively, and the value of the internal variable w ranges from 0–1. The nonlinear coefficient, denoted by α, adjusts for potentiation (α_p_) or depression (α_d_), with 1 being the ideal value. Further, the prepared synaptic transistor had nonlinearity coefficients of 1.56 and 0.16 for potentiation and depression, respectively. This study designed the synaptic weights of the ANN with extracted factors and normalized conductance from the fabricated synaptic transistor. Subsequently, the ANN was trained using approximately 60,000 MNIST simulation datasets, and the recognition rate was calculated by changing the number of hidden nodes from 10 to 300. Figure 8c shows the recognition rate according to the number of hidden nodes in epoch 1. Although the recognition rate in 10 hidden nodes was 74%, with an increase in the number of hidden nodes, a sufficient recognition rate higher than 92% was obtained.

## 4. Conclusions

This study proposed a biocompatible polymeric electrolyte-based EDL synaptic transistor using a PVA:CS mixed solution with high ionic conductivity. The EDL effect of the PVA:CS blended electrolyte used for the gate insulator of the synaptic transistor was verified through frequency-dependent capacitance characteristics. Consequently, the PVA:CS blended electrolyte exhibited EDL behavior owing to high capacitance and abundant internal mobile proton ions. Further, in the transfer characteristics of PVA:CS biopolymer blended EDL synaptic transistors, counterclockwise hysteresis owing to slow polarization reaction of mobile protonic ion migration was observed on gate voltage double sweep transfer curves. In addition, with an increase in the maximum gate sweep range, the hysteresis window increased in a linear manner, although the threshold voltage remained constant. Moreover, typical biological synaptic behaviors, including short-term and long-term plasticity and high-pass filtering capabilities, were demonstrated through single-spike, frequency-dependent EPSC, PPF, STDP, and potentiation and depression properties. Furthermore, through learning simulations on the MNIST handwritten digit dataset, an excellent recognition rate of over 92% was achieved, thereby validating the ability to efficiently mimic biological synapses. Therefore, PVA:CS biopolymer blended electrolyte-based synaptic transistors are expected to provide potential applications for building environmentally friendly and highly biocompatible neuromorphic systems in the future.

## Data Availability

Not applicable.
